# Developmental Ecological Psychology meets organicist biology: the example of the ecological self

**DOI:** 10.3389/fpsyg.2025.1569356

**Published:** 2025-07-01

**Authors:** Catherine Read, Agnes Szokolszky

**Affiliations:** ^1^Rutgers University, New Brunswick, NJ, United States; ^2^Ithaca College, Ithaca, NY, United States; ^3^Institute of Psychology, Szeged University, Szeged, Hungary

**Keywords:** development, Developmental Ecological Psychology, ecological self, ecological-organicist theoretical framework, organicist biology, James Gibson

## Abstract

In this paper we outline an ecological-organicist theoretical framework to understand human development. The ecological approach to development (Developmental Ecological Psychology, DEP) places the organism at the center and has a mutualist theoretical framework with an epistemic foundation in direct perception. While research in this tradition has paid much attention to specific developmental questions at a young age (such as perceptual learning, affordances, and action coordination), less effort has gone into the discussion of theoretical issues of overall development at the level of organism-environment mutuality. Meanwhile in biology, a new emphasis on the organism as an explanatory concept and level of analysis has been asserted (e.g., Nicholson, 2014). In this paper, we are seeking possible fruitful ideas at the intersection of the ecological approach and the renewed organicist thinking in biology. We suggest that organicist ideas are relevant for an ecological theory of development and the epistemic foundation of direct perception is important for a consistently mutualist organicism. We examine Waddington’s epigenetic landscape model and Gottlieb’s probabilistic epigenesis from an ecological-organicist point of view and suggest, in contrast, a consistently ecological-organicist approach to self, i.e., the ecological self, based on J.J. Gibson’s idea of co-perceiving self and surround.

## 1 Introduction: Developmental Ecological Psychology meets organicist biology

James Gibson’s ecological approach to perception (1966, 1979) is radical in centering the animal (and human) *organism* as the one who perceives/acts, and in describing the organism and its surround as existing mutually, making direct perception of the relevant aspects of its surround possible. We take the centering of the animal and human organism as our starting point to elaborate developmental processes of perceiving/acting/knowing. Further, we draw on recent work in developmental biology and the philosophy of biology that also centers the organism, to lay the Developmental Ecological Psychology foundation for a discussion of the development of the ecological self (see Gibson, 1979; Grene, 1993). Reciprocally, we also point out that direct perception is a resource for developmental biologists who endeavor to elucidate the actual contact of the organism with its surroundings (Read and Szokolszky, 2024; Costall, 2001).

In this paper, we continue conceptualizing the ecological approach to development, which Eleanor J. Gibson and her followers substantially developed (see, e.g., Gibson and Pick, 2000). We suggested that Developmental Ecological Psychology (DEP) is an emerging discipline itself, and as such, it must enlarge its agenda and address broader problems of development (Szokolszky and Read, 2018). We also raised the question of how Ecological Psychology can become more developmental and how DEP can become more ecological. We observed that research in DEP has focused on the differentiation of perceptual systems as a basic process, but the relation of perceptual systems to whole organisms existing in mutuality with their surroundings is yet to be elaborated (Read and Szokolszky, 2018c, 2024).^1^

In his ecological epistemology, J.J. Gibson placed the organism at the center of research by suggesting that direct perception of what the surrounding layout (including other organisms) affords, i.e., perceiving/acting, is the foundation of all psychological functioning. In Ecological Psychology the organism is an active agent who is in direct epistemic contact with its environment (Gibson, 1979). Direct perception is essential for a consistent organicist theory because it is the key to a non-dualist relationship between “the knower and the known” (using Dewey’s and Bentley’s expression, 1949). Theories of indirect perception assume that perception is based on meaningless sensations, enriched by cognitive operations. The age-old idea implies animal-environment dualism—the logical separability of animal and environment and an “animal-neutral environment.” Once these assumptions are taken, mechanisms need to be assumed that bridge the presumed gap between the animal and the meaningless, animal-neutral environment (Turvey and Shaw, 1979). Ecological psychology, however, postulates the evolutionary and logical necessity of non-dualism and the ontological and epistemological interdependence of the animal and its environment. The animal evolved to search and find aspects of the environment that are directly meaningful for their interests and survival. Perceptual systems evolved in an environmental niche where the animal and the environment have been constantly and mutually tailored to each other (Gibson, 1966, Michaels and Carello, 1981). On this line of thought organicist theory must rest on the assumption of direct perception in order consistently to embrace organism-environment mutuality and avoid the consequences of dualism.

Early developmental work in perception from an ecological point of view focused on learning—specifically, differentiation and integration—of perceptual features and the conditions that facilitate or interfere with this learning in human infants (see Gibson and Pick, 2000, for a review of the literature). Research on social interaction has focused on rhythms and changes in rhythms in the interactions, again, with a focus on learning (Adolph et al., 2010). Movement research with infants used a dynamic systems analysis focused on behavior broadly defined as changes in the “motor system” that allowed different interactions with the environment (e.g., Thelen and Smith, 1994) or defined as adaptive coupling to the environment, including other people (Rączaszek-Leonardi et al., 2022). These fruitful and systematic research programs have greatly added to the field of DEP and developmental psychology at large and continue to do so. But key questions are: what characterizes and distinguishes the life course of organisms, especially human organisms, from other kinds of organisms and nonliving systems? What are the consequences if the organism is taken as the primary starting point in developmental and perceptual research and theory (rather than ‘behavior’ or ‘systems’)?

Two innovations, one in psychology and one in biology are now beginning to affect the field of developmental psychology. The first is James Gibson’s effort to describe and study direct perception (as opposed to the functioning of the senses) at the level of the organism as an agent (cf. Read and Szokolszky, 2020; Heft, 2020). The second is the new emphasis on the organism as an explanatory concept and level of analysis in biology (e.g., Nicholson, 2014). It has been argued that the most important tradition within early twentieth-century philosophy of biology was the organicist movement which has clear connections to present-day issues related to the nature of living systems (Nicholson and Gawne, 2015). In the present paper, we are putting forth an *ecological-organicist approach* to development, seeking to find possible fruitful ideas at the intersection of the ecological approach and organicist thinking in biology. We argue first, that organicism can be a resource for ecological developmental theory, and, second, that the epistemic act of direct perception is essential for a consistent organicist theory.

In what follows, we examine the concept of development in two influential theoretical approaches: C. H. Waddington’s “epigenetic landscape” model (Waddington, 1942, 1957), and Gilbert Gottlieb’s probabilistic epigenesis (Gottlieb, 2007). These theoretical models present systems approaches that are effective in going beyond earlier mechanistic concepts of development, but they do not make the organism primary and they lack an epistemic foundation based on the mutuality of animal organism and surround. We discuss renewed organicism in developmental biology and delineate some consequences for theories of development if perception is seen as direct resonance to the structure of the surroundings, as opposed to the functioning of the senses. We also explore the consequences of taking the organism as the organizing concept. In the final section examine how these ideas can be applied to a specific phenomenon, the development of the ‘ecological self’.

## 2 Concepts of development in biology and psychology

Concepts of development in biology and psychology traditionally differ in focus. In biology development primarily refers to the development of the embryo to full differentiation of tissues and organs, whereas in psychology and comparative psychology, development generally means changes or transformations taking place for some time after birth or hatching (although pre-birth experience is also studied in relation to later development). These two strains of developmental thinking influenced each other. In 1966, while Schneirla (1966) was arguing that development is key to understanding animal behavior and evolution, Gibson was working on the relation of animal organisms to their surroundings through direct perception (Gibson, 1966). Although mainstream comparative and developmental psychology retained the assumptions that perception was just the functioning of sensory organs (with added neural ‘processing’), Gibson worked out a radically different approach to perception based on the mutuality of organism and surround (Gibson, 1979; see also Costall, 2001, Read and Szokolszky, 2024). These two research threads both engendered early developmental research in comparative psychology and human development. Research from the ecological approach to perception concentrated on the earliest beginnings of human and animal life, not to test what was instinctive (cf. Schneirla, 1966, p. 288), but to show early and early developing perceptual activities (e.g., Gibson and Pick, 2000).

At the same time that Comparative Psychology and Developmental Psychology were becoming recognized fields of study, biologists were working on an approach to living organisms that was neither mechanistic nor vitalistic, thus constituting the early examples in modern science of organicist thinking.

### 2.1 C. H. Waddington’s “epigenetic landscape”

During the 1930s–40s, Conrad H. Waddington, along with other scientists and philosophers gathered in the Theoretical Biology Club and interested in embryology, were trying to chart a “Third Way” departing both from mechanists and vitalists.^2^ Representing a new kind of organicism, they aimed to develop an understanding of the organism as defined by process and organic wholeness, as an alternative to mechanistic explanations. From Gestalt psychology, the group accepted the guiding idea that the whole is different from the parts and that the parts determine the whole as much as the whole determines the parts. They added that process is more important than individual particles, and also adopted principles from emerging systems theory and claimed independent laws for biology without leaving scientific ground. At that time the promise of an emerging molecular biology was seen as a way to bridge the gap between biology and physics by offering biochemical explanations for self-regulatory developmental processes in the organism (Dresow, 2017; Chichester, 2023).^3^

**Figure 1:**
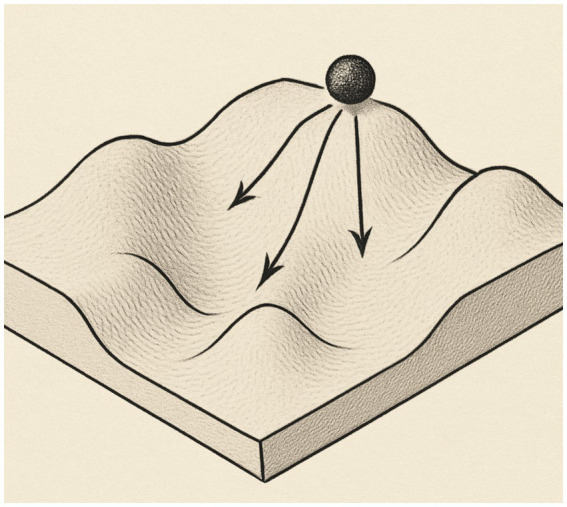
Waddington’s landscape model of development.

The new view of biology required new images and C. H. Waddington famously used the landscape image to guide thinking about embryogenesis, naming the process ‘canalization’ (see Figure 1). He introduced the term ‘epigenetics’ into biology in 1942 (Waddington, 1942) and saw the organism as developing in an “epigenetic landscape,” a visual model of forces that channeled cell differentiation at certain points in embryogenesis (e.g., Waddington, 1942, 1957).

This visual model implied the familiar experience of water flowing due to gravitation and that the differentiated contour of the landscape shapes the “flow” of development from fertilized ovum to mature form. It was meant to define a whole set of complex developmental processes between ‘genotype’ and ‘phenotype’. The ‘genotype’ underlay and formed the surface, and the cell/organism could only adapt to the environment, i.e., roll downhill. In this model, Waddington coordinated several ideas, namely “that the embryo’s parts (i) are in dynamic disequilibrium (like water running downstream) with a progressive loss of potential, (ii) follow a developmental track which, as a whole, is more or less stable…, and (iii) generally decrease their sensitivity to disturbances, from periods of high sensitivity where regulation is possible (‘a valley with gently sloping sides’) to periods of strong canalization (‘the valley as having vertical sides’)” (Fusco et al., 2014, p.118). These ideas are very similar to those proposed for the development of animal *behavior*, not just *form*, by early animal behavior biologists and comparative psychologists (e.g., Schneirla, 1956). The ideas, if not the visual representation, of an organism drawn down on a “landscape” of forces transferred into traditional developmental psychology at about the same time (see Cairns and Cairns, 2007).

The landscape image is still used in biology as well as in psychology, although it is very limited in the capacity to portray the interaction of organism and surround. In biology, the landscape image is most often used for studies of cell differentiation, but cell differentiation cannot be assumed to cover the full range of dynamics of other developmental processes “like cell proliferation, movement and death, production and consumption of extracellular material, morphogenesis, pattern formation, and growth” (Fusco et al., 2014, p. 126). And, of course, any behavioral study of development must include, beyond growth, the processes of perceiving, learning, and experience (cf. Szokolszky et al., 2019).

In short, the landscape image is too mechanical to underpin the development of organisms that perceive and act. In Waddington’s model, a ball passively rolls downhill due to gravity—that is, ideas from mechanics took over from ideas of living organisms (Read and Szokolszky, 2018c). Even the geophysical processes of how the landscape was formed (e.g., forces such as cooling/heating, evaporating/condensing) were left out of the picture. The canalization model is a relatively direct ancestor of dynamic systems models, which are based on state changes in thermodynamic physics and are currently used in developmental psychology (Thelen and Smith, 1994; Smith, 2006; Muchisky et al., 1996) and in the Ecological Psychology of adult action (e.g., Zaal and Bootsma, 2000). (See Baedke, 2013, for an analysis of the current use of canalization ideas in various scientific fields see Deichmann, 2016).

### 2.2 Gottlieb’s probabilistic epigenesis

An influential use of the idea of epigenesis was developed by Gilbert Gottlieb and associates beginning in the 1970’s. Gottlieb is recognized as a central figure in introducing developmental thinking in psychology by exposing the sterility of the nature–nurture debate and the adoption of a biological framework that conceives of living entities as self-organizing systems. The themes central to his conception of epigenesis included the notions of differentiation and functional integration, bidirectionality between structure and function, and the fact that new information for development is generated by the process of development itself (Gariépy, 2007). Gottlieb also presented his theory visually, and like Waddington’s, his depiction also became influential. The model depicts four parallel lines labeled from the bottom “Genetic Activity”, “Neural Activity”, “Behavior”, and “Environment (Physical, Social, Cultural),” with the label “Individual Development” which progresses from left to right (in time). Between each pair of lines are arrows indicating interactions between each pair of levels.

This stratified, linear model divides organisms into levels, that is, genetic, neural, and behavioral, and then adds the level of the ‘environment’ (everything outside the organism that it is in contact with). The model allows for bidirectional effects between contiguous levels and, in addition, makes the interactions probabilistic. A recent explication places these ideas in relation to systems approaches in biology and psychology (Gottlieb et al., 2006). The probabilistic epigenesis model of development, and its system extension, has been very effective in countering the old, but also ongoing (Armstrong-Carter et al., 2021), emphasis on the causal role of ‘genes’ in ontogeny. Working from Schneirla’s emphasis on maturation and experience, as opposed to ‘nature’ or ‘nurture’ (Schneirla, 1966, p. 288), the epigenesis model significantly extended research and thinking on the relationship between ‘genes’ and ‘environment’. Specifically, the approach emphasizes the *coaction* of genes and environment, rather than positing interaction. The idea of the coaction of genes and environment implicates the interconnectedness of gene–environment relations in individual development. Specifically, all developmental outcomes are the result of genes operating in a particular developmental context, and outcomes are likely to change when the context changes (Gottlieb, 2007, p. 6).

For our present purposes, it is important to highlight several aspects of this model. First, this model is linear in time and in ‘layers’, even though effects can be either upward or downward between contiguous layers. We ask: Where is the organism in this model? The boundary between ‘behavior’ and ‘environment’ is no different than the one between ‘behavior’ and the ‘neural system’. This results in some odd phrases such as “genotypes can be reared in different environments.” Clearly, genotypes are not reared, and the author did add in parentheses “actually organisms” (Gottlieb, 2007, p. 7), acknowledging implicitly that the organism is the subject of a theory of development. In fact, the organism is left out of the model.

We discussed Waddington’s and Gottlieb’s models to make the point that while these influential models represented important steps toward a “more organicist” understanding of development compared to traditional views, they remained limited in their conception of a developing organism embedded in its environment in a mutualist way. Importantly, they also retained the idea of indirect perception, that is the old idea that contact with the world consists of the functioning of sensory organs (cf., Read and Szokolszky, 2020). Thus, leaving developmental processes without firm ontological and epistemological foundations. Next, we argue that recent efforts toward organicism, when combined with ecological direct perception, show promise for a profoundly mutualist conception of development. We delineate the idea that the organism is a primary unity existing in constant reorganization throughout its life cycle, which necessarily includes development. Our main claim is that the animal and human organism is always an ecological self in the sense that it is a self-perceiving, transforming and developing agent, the originator of its movements and actions.

### 2.3 Centering the organism: a proposal for an ecological organicist approach to development

The twenty-first century has seen a resurgence in the biology of organicism which was originally conceived to counter mechanistic reductionism in explaining living systems while remaining materialist in its theoretical explanations (cf. Gilbert and Sarkar, 2000; Gilbert and Bard, 2014; Nicholson, 2014; Nicholson and Gawne, 2015). The idea that living systems contain different levels of functioning that affect the system both from the bottom up and from the top down is key to the organicist approach, along with a non-reductionist ontology and explanations. As explained by Gilbert and Sarkar (2000, p. 2), reductionist ontology would see a tissue as an organized collection of cells and cells as an organized collection of organelles (and so on), organicist ontology, along with bottom-up considerations would also include the functioning of the tissue within the organism, the functioning of the organism within the environment (and other parameters as well). “The structure and function of a hepatocyte depend not only on the properties of organelles comprising it but also on the properties of the organ in which it resides…. The properties of any level depend both on the properties of the parts “beneath” them and the properties of the whole into which they are assembled.” (Gilbert and Sarkar, 2000, p. 2). In addition, forms or structures are emergent, that is, properties are emergent if their presence cannot be explained based on the constituent parts. And every “level of organization has its own regularities and principles, not reducible to those appropriate to lower levels of organization, nor applicable to higher levels” (Gilbert and Sarkar, 2000, p. 3).

What are some of the consequences of the organicist approach to theories of biological development? In the case of organisms with differentiated gametes, the fertilized egg inherits DNA, it does not inherit ‘genes’. Genes and gene products are constructed anew in each cell of the developing embryo. The gene is a higher-order structure than the DNA, and gene construction is affected by an even higher-order structure, the cell (Gilbert and Bard, 2014, p. 130). Thus, “the relationships between cell surfaces generate morphogenetic fields, tissues, and organs. The body builds itself as it develops, each whole becoming part of something larger that it generates, and each whole defining the context of its parts. Development is a creative choreography of molecules, cells, tissues, organisms, and ecosystems” (Gilbert and Bard, 2014, p. 141) and each organism is a new ‘developmental performance’ (ibid.). The emphasis on wholes at different levels of existence is organicist, but note that the organism, on this account, is not a privileged level—it is equivalent to the levels ‘below’ and ‘above’ it.

What if the organism is taken as the starting point for developmental biology and developmental psychology?^4^ Nicholson (2014) points out that questions regarding the nature of organisms, such as how they maintain their organization and coordinate their functions in a way distinct from inanimate systems, have been absent from biology and the philosophy of biology since the mid-twentieth century. But if the organism can once again be a focus of biology, there are certain consequences for theory and research. We note these consequences here as we will build on them when we discuss the psychology of developing organisms.

Organicism in developmental biology is a potential resource in thinking about developing organisms. Organicism in general is an approach in biology that takes the organism as a level of analysis, as opposed to investigating only the molecular or species levels (cf., Nicholson, 2014; Walsh, 2015). But what is an organism? And what are the consequences of not only recognizing organisms as a level of biological existence but of taking organisms as the primary level? At what level does autonomy and/or agency come into biology (cf., Moss, 2024; Rosslenbroich, 2014, 2023; Read and Szokolszky, 2024)? We will use the definition of organism that was presented by Webster and Goodwin (1996) in an early line of argument centering on the organism in biology:

“A machine, said Kant, is a functional unity in which the parts exist for one another in the performance of a particular activity. An organism, on the other hand, is both a functional and a structural unity in which the parts exist for *and by means of* one another. The distinguishable parts of an organism (leaves, flowers, limbs, eyes, etc.) do not preexist before being assembled into a functional whole, as do the parts of a machine. Rather they emerge from the interaction of spontaneously generated differences that give rise to parts within a primary unity. This unity persists throughout the generative process and into the form that we recognize as a mature organism of a particular species. In fact, it extends beyond the mature form into the next generation via the gametes, parts of the organism with the capacity to produce new wholes, since the organism of which we speak as the fundamental entity in biology is a life cycle.” (Webster and Goodwin, 1996, p. 193, emphasis in the original).

Two aspects of this definition are important for our argument about organisms in biology and developmental psychology. First, the idea that the organism is a ‘primary unity’ and the second that the organism is a ‘life cycle’, which necessarily includes development. If the organism is seen as the cause, or at least, the means of its own unity, and if that unity is a life cycle (that includes ‘birth’ and ‘death’), then what is unique to that type of unity? Organisms have life cycles and metabolisms: these qualities apply to all organisms, including plants and protists. But when we come to qualities such as ‘activities’ and ‘choices’ (Nicholson, 2014, p. 350), we as ecological psychologists, claim that these qualities apply only to animals and humans (Read and Szokolszky, 2024). J. J. Gibson began with the animal as the one that perceives/acts, implicitly including human beings. In his work on visual kinesthesis (1979), he included animals with humans in the phenomenon of co-perceiving self (body) and surround and even claimed that this meant that the animal was self-conscious. We would amend that statement to say that the animal is ‘self-aware’, at least of self as a body.

A further consequence of centering the organism in biological thinking is that evolution is seen not as a function of genes (transmission, selection, variation in the population), but, instead, inheritance, development, and adaptation are unified by the capacities of organisms, such as their plasticity and robustness, and, we would add for animals, their capacities for autonomy (cf. Rosslenbroich, 2014, 2023). Organisms, therefore, are the primary agents of evolutionary change (Nicholson, 2014, p. 349). And, further, evolution depends on development, and development depends on the functioning organism in its surroundings. We note here that such qualities as plasticity, robustness, and autonomy *depend on* a non-dualistic direct contact with the surroundings by the organism, that is, direct perception.

If organisms are active, through their metabolisms, their activities, and their choices, then they do not passively adapt to pre-existing ‘environments’ as is assumed in the Modern Synthesis view of evolution (Nicholson, 2014, p. 350). Certainly, all living organisms embody processes of respiration, metabolism, and reproduction. But the living body also dies, that is, it decomposes. This directly observable quality of living organisms distinguishes them from non-living systems (cf., Plessner, 2019). Mechanical systems dissipate, they do not decompose. Volkswagens disintegrate, they do not develop into old age. Not all organisms, however, have activities or make choices; these latter qualities belong exclusively to animals and humans (e.g., Read and Szokolszky, 2024).

#### 2.3.1 An ecological-organicist view of development

Before one can describe/explain how development takes place, one must characterize *what* it is that develops. Various current theories of development give very different accounts of the ‘what’, of what develops. Organismic relational theory focuses on the development of action (e.g., Raeff, 2016) over the lifespan. Dynamic systems approaches to development center the movements or actions that are best described with dynamic systems mathematics, that is, in terms of how different variables of movement form a changing but coherent system (e.g., Thelen and Smith, 1994) at any point in development. Development here is defined as time-dependent changes in behavior (Muchisky et al., 1996). Epigenetic developmental theory draws on Neo-Darwinian evolutionary theory, focuses on comparative development, and on species-specific behaviors that develop at certain times in the animal’s life, and through proposed coaction of genetic and environmental factors (e.g., Gottlieb, 2007). Developmental work in line with Ecological Psychology has focused on behaviors that indicate perception in early infancy concerning objects or other people (Gibson and Pick, 2000), and posited that the main processes of development are differentiation and integration of perception. In this case, what develops are the perceptual systems of an exploratory organism.

At this point, we emphasize the intersection of organism-centered biology with J.J. Gibson’s ecological approach to perception. Gibson centered the animal organism as the organism capable of perceiving and acting. His ecological approach entailed a critical distinction between the functioning of the sensory organs and actual perception. This distinction is made by no one else in biology or psychology, but it is critical to understanding the relation of the animal organism as mutual with its surroundings (e.g., Costall, 2001).

We propose to build on the idea of perceptual differentiation/integration, by describing the development of animal and human organisms that are always active *agents* existing in mutuality with their surroundings *because* of their abilities to perceive directly the layout of surfaces surrounding them as they act. In other words, we claim that perceiving is always agentive because the acts of animals are agentive, and perceiving is always an act (cf. Read and Szokolszky, 2024). Specifically, we propose that the living animal organism in mutuality with its surroundings is the starting point of development (as opposed to DNA, genes, or cellular functions; or organisms that have to adapt to pre-existing environments) and that as active agents animals are always *perceptible to themselves*, distinct from their surroundings (Gibson, 1979; Read and Szokolszky, 2020; Read, 2024).

For human beings, and some types of animals, life takes place in a social context—individuals exist in relation to other individuals (conspecifics and allospecifics). Self-initiated agentive action, then, can be toward or with, or against other agents. And to the extent that humans or animals have modified their inanimate surroundings, individuals live with the effects, extended in time, of agents’ previous actions. They exist in a socio-cultural surround.

Given that the starting point for an ecological approach to vision was the animal organism in mutuality with its surroundings (Gibson, 1979), the question of what develops is answered: it is the organism that develops, always in mutual relation to its surround.^5^ The organism moves, acts, learns, and develops, all in the context of directly perceiving and acting in the surrounding layout, which includes other agents and traces left by other agents. The fact of mutuality does not change throughout the lifetime, and the surroundings do not develop, although they change, either by nature or by human construction. The organism is always an ecological self in the sense that it is an agent, the originator of its movements and actions. It is a constant but also transforming, and developing. (We define agency broadly here, in contrast to definitions that depend on invoking knowledge (e.g., Keijzer, 2021; cf. Read and Szokolszky, 2024).

What are the consequences for developmental theory of taking the organism-in-mutuality as the starting point? If we limit ourselves to animal organisms because we are interested in those that can perceive/act, in other words, to those that have an ecological self, then we focus on the development of that self.^6^ And that development involves reorganizations in what is the unity of the organism and the unity of the organism’s experience over time. That is, if development originates with the organism, then it is broader than processes such as learning and experience, and it subsumes them. Below we present ways of visually representing development that are consistent with direct perceiving/acting, that is, the mutuality of animal and human organisms with their surround.

#### 2.3.2 Visual representations of an ecological—organicist view of development

**Figure 2:**
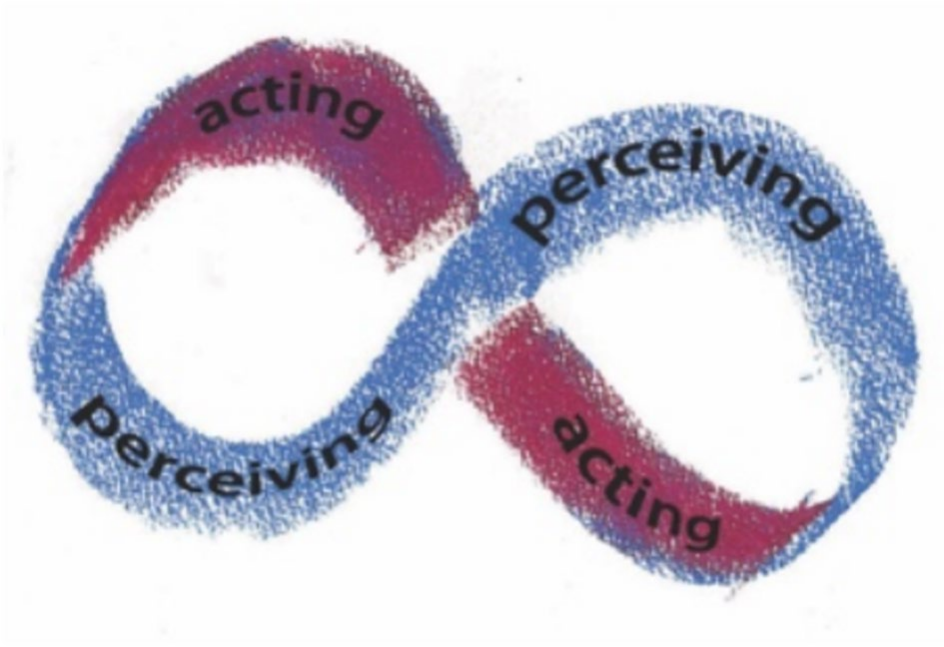
An ecological diagram of perceiving/acting.

Figure 2 presents the relation of perceiving and acting using a Mobius strip image to emphasize that perceiving is always acting, and acting always changes perceiving—what is perceived and how it is perceived. This image pertains to the ongoing activities of an exploratory perceiving organism, but it does not indicate what *development* of the organism would look like.

**Figure 3:**
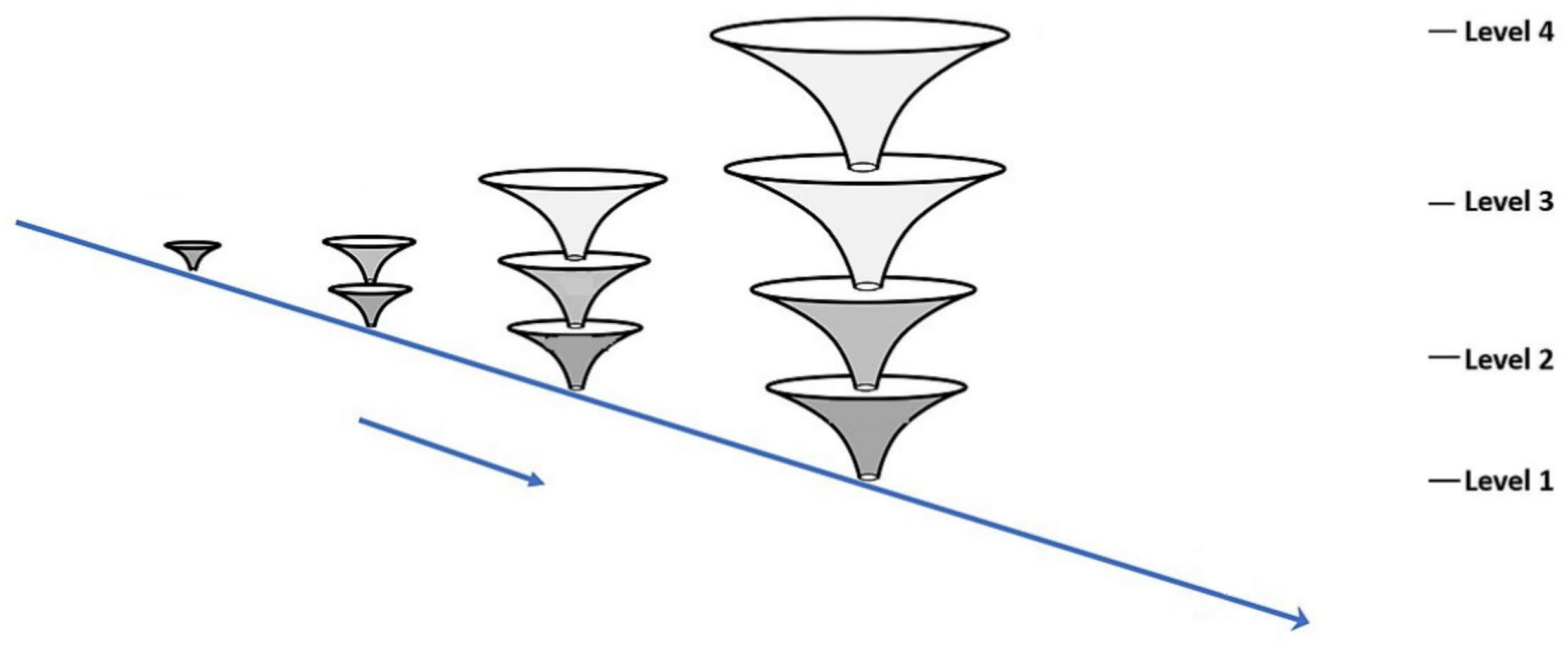
An organicist-ecological diagram of development.

Figure 3 indicates a ‘punctuated’ development model in which qualitatively different organizations of the organism arise from the previous organization in steps or stages.

This diagram does not indicate a full life course, rather it shows how development proceeds in its duration over several stages of reorganization. This is a punctuated view of development and focuses on emergent transformation. If we apply these ideas to human beings, for the moment considering only human development, the description could apply from the single fertilized cell to the multicellular organism, to the emergence of tissues and organs, and so forth, until birth. At that point, the infant emerges from a watery world into a world of air, solid surfaces, and weight. The infant continues to see, hear, and act after birth, but now the surroundings are radically transformed, and the infant’s perceiving and other activities are transformed in their continuing mutuality with the physical and social surround. For example, the social surround now becomes visual as well as auditory, and it becomes tactual—the infant can see touch, and feel the people around her. Perhaps the next to emerge is perceiving in a focused and controlled way, as many studies of infant perceptual activity have shown (e.g., Bahrick et al., 2002). The challenge to the developmental psychologist is to discern transformations or reorganizations of the organism at the behavioral/activity level, as well as at the biological level. We note that this model does go beyond the typical biological or comparative model (see above on Waddington and Gottlieb) which includes a ‘line’ between the ‘organism’ and the (abstract) ‘environment’ that is not directly bridged in the accompanying theory and explanation.

**Figure 4:**
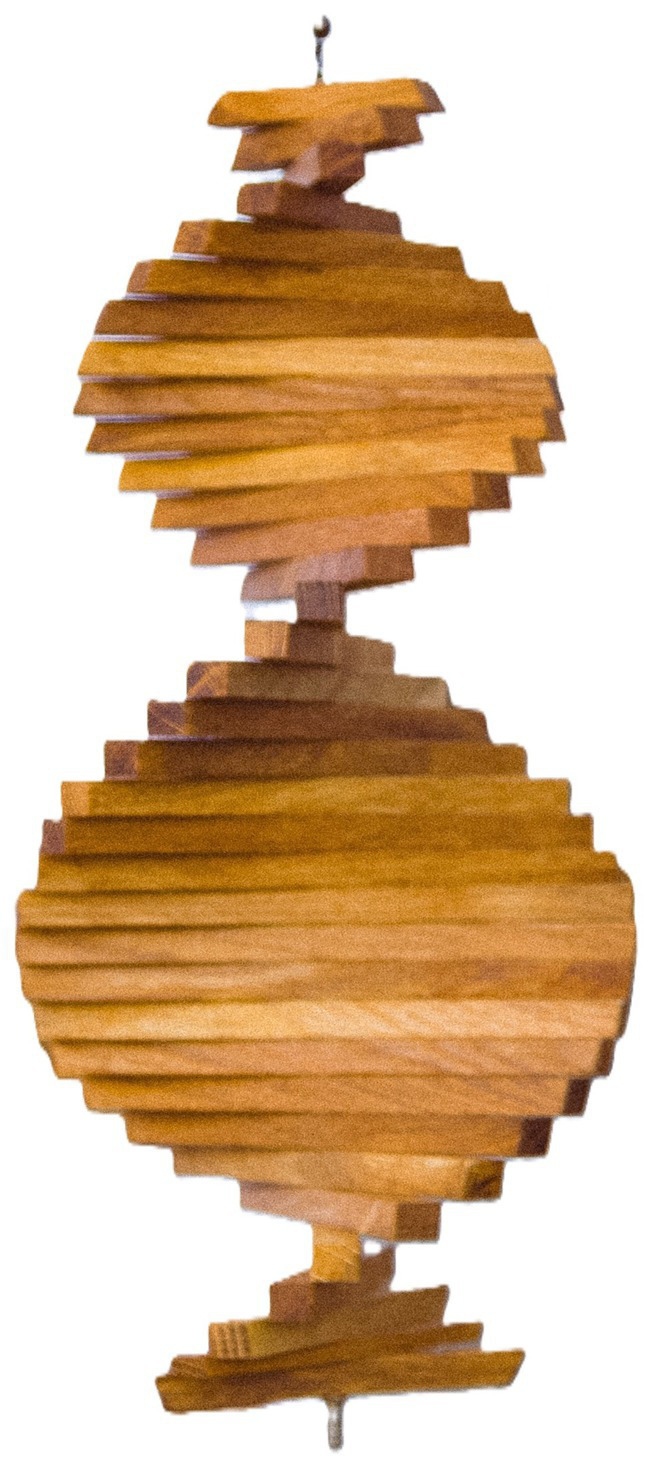
Three-dimensional spiral.

In a further step, if we make the model three-dimensional as in Figure 4. We then present the full life course as a spiral extended in duration with the ‘saddle’ geometric formation of two simultaneous and complementary movements. The image below illustrates a rotated level plane that results in curves that simultaneously, that is in one form, curve upward/outward, and downward/inward resulting in a form such as that of the saddle for holding a rider on a horse’s back. This image can aid in thinking about the outer and inner aspects of the organism-in-surround. Animals, inasmuch as they can initiate action, have an inner aspect. In human beings, this inner aspect becomes highly developed by adulthood. But animals and humans always live in and with their surroundings, which are the outer aspect.

This model goes far beyond the idea of the ‘interaction’ of organism and environment (cf., Gottlieb, 2007; Oyama et al., 2001). The moving spiral model, instead, is consistent with *an organism in mutuality with its surround*, that is, as always directly perceiving/acting in and on the layout of surfaces, and the other organisms, around it. The model is also *developmental*. There is a progression in the spiral, an expansion, and then a contraction. The animal or human organism and its possibilities for action, activity, acting, and reacting expand to the mature form, and then begin to lessen toward the end of life in a full life cycle.^7^ This organicist developmental approach contrasts with traditional theories of development in that it does not focus on the development of specific abilities, behaviors, or capacities, but, rather, on the development of the whole organism in which capacities ‘reside’. The organicist developmental approach requires a different kind of thinking than previous theories. It is a kind of thinking that is consonant with the mutuality of organism and surround through direct perception that James Gibson spent his academic life propounding and refining.

**Figure 5:**
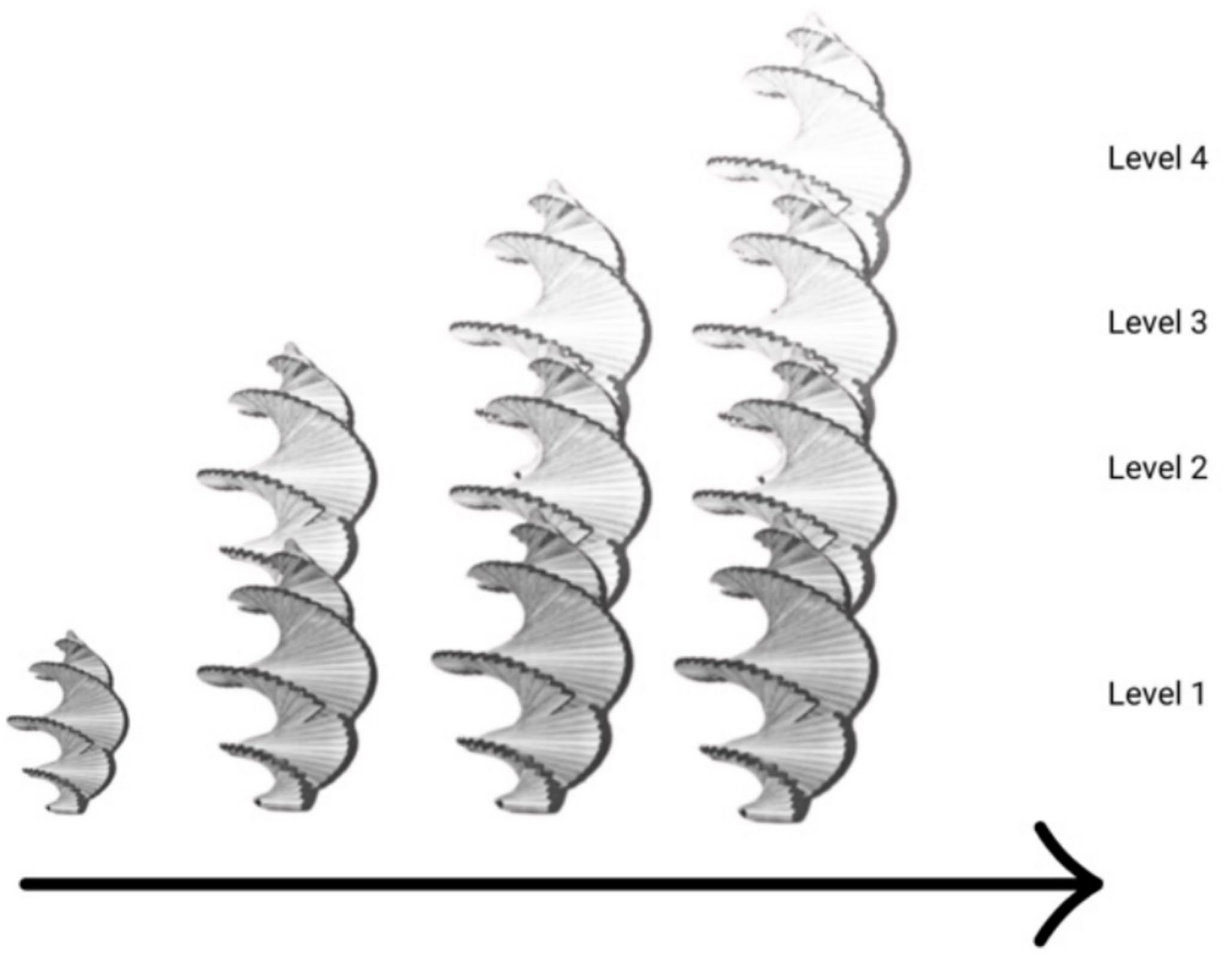
Progressive levels of development as spiral functions.

The spiral captures the phenomenon of rhythmic change over time with expansion and contraction. It does not capture the phenomenon of reorganization or transformation. If we combine Figures 3, 4, we have something that combines the spiral form with reorganization: emerging spirals over the course of development (see Figure 5). We will draw on this latter form to discuss the development of the ecological self.

## 3 Organicist Developmental Ecological Psychology: the example of the ecological self and its development

Gibson (1979) characterized visual kinesthesis, the simultaneous co-perceiving of self (body and other aspects of self) and surround as the basis for self-perception. We take this phenomenon as a core example of the concept of the “ecological self” and agency in direct perceiving/acting, and herein expand this idea in line with organicist thinking on development. Our goal is to show that organicist developmental thinking can support and further develop our understanding of the ecological self. And, in turn, understanding the ecological self will result in a more comprehensive Developmental Ecological Psychology. The self should not be ignored.

### 3.1 J.J. Gibson’s foundational idea concerning the self: co-perception

In his last book, The Ecological Approach to Visual Perception (1979), J.J. Gibson included a chapter on “The optical information for self-perception.” He explained that his “field of view” concept specifies the environment, as well as the self-embedded in the surroundings, like two sides of the same coin. The field of view is the combined fields of the two eyes from a specific point of observation occupied by the perceiver. This moving point of view specifies both the surround and the perceiving agent—the embodied ego. By specificational information, Gibson meant direct perception, which is representationally unmediated, and directly meaningful. He framed his approach to vision based on the idea that the organism with a visual system resonates to structure in the ambient array that specifies aspects of the surrounding layout of surfaces due to the mutuality of the organism with its surround such that the organism and its surround imply and co-define each other (Michaels and Palatinus, 2014; Read and Szokolszky, 2020, 2024).

Regarding self-perception, Gibson laid out the basic idea of co-perceiving: “The optical information to specify the self, including the head, body, arms, and hands, *accompanies* the optical information to specify the environment. The two sources of information coexist” (Gibson, 1979, p. 116). “One perceives the environment and co-perceives oneself” (ibid. 126). This seemingly simple observation has profound consequences for a notoriously difficult concept in psychology: the self. Once we assume that the self and the surrounding environment are continuously co-perceived in the flow of direct perception, the idea of the self as a subjective mental state hidden inside evaporates. “The experience of a central self in the head and a peripheral self in the body is not, therefore a mysterious intuition or a philosophical abstraction but has a basis in optical information,” as Gibson put it (ibid. 114). From the inseparability of ego perception and exteroception, it follows that “the “subjective” and the “objective” are not separate realms but only varying poles of attention” (ibid, 116). Thus, attention is the manifestation of agency.

Gibson described examples of the co-perceiving of self and surround; how the human body protrudes into the optic array, referring to body parts, such as the end of the nose and the lower part of the arms with the hands as “subjective objects.” He used this term to emphasize ways in which such visual arrays cross the objective-subjective divide. This is a consequence of the mutuality principle, according to which the organism-and-its-surround is the irreducible unit of analysis. In his 1979 description, the perceived self mostly came up in the context of the moving body. He discussed the continual lawful changes in the optic array depending on the organism’s movement and the phenomenon of perceiving the value or affordances of the objects and surfaces in the layout. However, he meant more than just bodily self-perception: “Ask yourself what it is that you see hiding the surroundings as you look out on the world—not darkness, surely, not air, not nothing, but the ego!” (Gibson, 1979, p. 112, cited by Costall, 2001, p. 481).

Gibson did not go on to explicate how the perceived ego is self-perceived agency. However, he established a new starting point for approaching the self from his ecological theory by showing that it is rooted in direct perception which is always dynamic co-perceiving of self-in-its-surround. Thereby he introduced the concept of the ecological self as an alternative route to the understanding of the self. The self is not based on a collection of self-related representations and cognitive constructions, but on the ever-present co-perceiving that presents a noncognitive foundation for self-awareness. It is a subjective awareness only inasmuch as the subjective and the objective aspects are varying poles of attention.^8^

E.J. Gibson (1993) on the development of exploratory perception stated: “The unmoving persisting world is continuously revealed in this streaming vista, the moving self and the unmoving world being reciprocal aspects of the same perception” (ibid. 26). She explained the early ontogenesis of the ecological self in infancy, using the processes of differentiation and integration. However, she also seemed to foreground bodily self-perception. In her description, the ambient optic array is structured by the surfaces surrounding an active organism, including the segments of its own body which form a “unique segment of the array” (Gibson, 1993, p. 26) which is the self.

Important new steps were taken by the Gibsons but much has remained unexplicated. First and foremost, the *unity* of the organism and aspects that go beyond the body per se have not been researched. They both discussed attention and perceiving the value of affordances of the environment, but that leaves questions regarding who is attending and who is valuing.

### 3.2 Ulric Neisser’s and Marjorie Grene’s expansion of the ecological self

In his widely referenced essay “Five Kinds of Self-Knowledge” Ulric Neisser (1988) drew importantly on Gibson’s ecological approach. He acknowledged that Gibson was the first theorist to insist that the self is “an inevitable counterpart of perceiving the environment” and that this idea is basic to the notion of what he called an “ecological self.” He claimed that “it is the whole person who perceives, acts and is responsible” (p. 3), nonetheless, he went on to argue that self-knowledge is based on distinct forms of information, each essentially establishing a different self. He defined the ecological self as the sense infants develop of their own physical body in relation to other objects in the physical environment and differentiated it from the “interpersonal self.” He claimed that the ecological self and the interpersonal self develop first in infancy and are then followed by the “remembered, private and conceptual selves,” arising later.^9^

In her essay on “The Primacy of the Ecological Self,” Grene (1993) took up the notion of the ecological self, but found Neisser’s treatment of it inadequate. She rejected the idea that the self directly perceived concerning the immediate physical environment can be equated with the ecological self, and, further, rejected the idea of separate selves. She also started from Gibson’s central thought of co-perception understood as direct perception: “To perceive oneself is, except in very peculiar circumstances, to co-perceive the world” (p. 112). In opposition to Neisser, Grene argued that aspects of the self cannot be separated. There are no different selves, “instead, I have access to myself under different aspects … I am a precipitate, so to speak, of a flow of events that both locate and define me and that, reciprocally, I have helped to shape through my own activity” (Grene, 1993, p. 112).

Grene pointed out that the self is more than seeing parts of one’s own body. She drew on Gibson’s delineation of perceiving as an activity in which the organism is in direct contact with its surroundings, but speaking of the ecological self, she defined it as an organismic unity in mutuality with its surround. Crucially, she emphasized that the self is a unity that exists over time. The ecological self can be studied at moments in time (as is typical in experimental work), but it persists over time. The human self usually persists over decades. It shows both signs of stability and instability, but throughout the lifetime the self can be described as “the pattern of the lived life from birth to death” (Grene, 1993, p. 114). By that, she arguably meant that the self is a continuously present and experienced ‘point of observation’ that distinguishes ‘me’ from the others.

Thus, Grene understood the ecological self as an overarching concept that incorporated its fundamentally social nature. She emphasized that for many animals, and certainly for human beings, the surroundings most often include other conspecifics. The surround of humans typically includes aspects that have been shaped by human beings in the context of their culture. Grene calls this a “culturally specific environment” (Grene, 1993, p. 114). According to Grene (ibid.): “the groups through which a self comes to itself make it the self it is, as it, in turn, helps to constitute the groups.” Self-knowledge is always in a place, both physically and socially. The ecological self, “who one is,” involves basically “where one is,” including shared inanimate and animate (social-cultural) surroundings populated by the conspecifics. That is, the self involves shared inhabitation, a “being-in-the-world” character with the consequence, as Grene points out based on Gibson, that only in a group can a person be highly individualized.

In relating self and self-conception, Grene drew on Gibson who stated that “knowing is an extension of perceiving” (1979/1986, p. 258). Gibson’s ecological theory closes the supposed gap between perception and knowledge. “To perceive the environment and to conceive it are different in degree but not in kind. One is continuous with the other,” he claimed and continued: “All knowing, perceiving as well as the most sophisticated scientific knowledge, in other words, consists in “an awareness of persisting structure” (ibid. 258). Grene posits that the same goes for self-knowledge, and ultimately for what we refer to as “the self.” “The self I recall, the self I know of as actor through time is… the ecological-social self, which from very early in its explorations of its world, has come to include cogitative … episodes …” (1993, p. 115). Grene concluded: “Thus, the self-knowledge that one is American seems to be built of layers of perceptual-conceptual awareness, some, but not all of which, can be made explicit. Just as perceiving and conceiving the environment are different in degree but not in kind, so are perceiving and conceiving oneself. The conceptual, social, and historical aspects of the self are “an extension or amplification of what is in the last analysis an ecological process with a structure that is best understood from an ecological point of view” (ibid., p. 117).

The point is that structure (of the self and the surroundings) has duration, even long duration, and it is perceptible and, thereby, knowable. The self is not something “inner,” or “secret,” but a public aspect of the personality that arises in group life longitudinally. Gibson points out that familiarity is a feeling that accompanies the perception of the persistence of some aspect of the perceiver’s surround, including other people (1979, p. 209). Persistence is perceivable.

### 3.3 The origins and development of the self, from the ecological-organicist point of view

J.J. Gibson’s foundational idea that perceiving is always co-perceiving, that is of surround and self-in-surround implied that the developmental origins of the self are to be found in the development of perception/action. This line of thought led ecologically oriented researchers empirically to delineate the origins of self-perception and the early events in the surroundings specifying the developing self. Already in the 1980s, ample evidence accumulated pointing out that an implicit sense of self is developing from birth, contrary to long-held views that children only begin to manifest self-knowledge by the second year (see the reviews, e.g., by Butterworth, 1992 and Rochat and Striano, 2000). Evidence from experiments and controlled observations has ever since grown to support the thesis that the origins of self are to be looked for in direct perception and action, as well as social transactions from birth on. The core theme is that the sense of the self emerges as the child directly and concurrently perceives its own body as it is acting and the particulars of the surround acted upon.

Immediately after birth neonates manifest haptic exploration via systematic hand-mouth coordination (Rochat et al., 1988). This exploratory behavior is already present in the womb, during the last trimester of pregnancy (De Vries et al., 1984). Early actions, like leg kicks, arm waves, and head turns produce an array of self-initiated visual-proprioceptive effects. Within hours after birth, neonates are capable of learning to suck in certain ways. Their sense of self-efficacy was demonstrated by experimental research. For example, as they discover that they can get a mobile strung overhead move by spontaneous kicking, they execute the movement with increased frequency to produce the result (Thelen and Fisher, 1983). Infants apply specific pressures on a dummy pacifier to hear another’s voice or see their mother’s face (DeCasper and Fifer, 1980; Walton et al., 1992). These observations (and many others) testify to the fact that early on infants manifest a sense of themselves as agents in the environment. We note here that the above research was carried out in the context of cognitive mental representation theory, so explanations included memory and mental representation. In contrast, we are working out an ecological approach, and provide a different account of the results. If one focuses on the activity of the whole organism in duration, i.e., on direct perceiving/acting, mental representation is obviated.

Infants can differentiate between the contingent live display of their leg movements and the noncontingent movement of themselves or another infant (Bahrick and Watson, 1985). They discriminate between self-produced and non-self-produced perceptual events. For example, within 24 h of their birth, newborn infants can discriminate between touching themselves and being touched externally (Rochat and Hespos, 1997). These are signs of self-awareness from the outset. Self-produced perception and action coupled with contingent proprioceptive perception specify the body as a unique entity differentiated from other objects in the environment. Self-specification is ubiquitous; in every action, the infant is presented with metamodal (visual and proprioceptive) information specifying the self.

From birth on, perception is developing as inherently social, as proven by extensive research in the past four decades. Earlier assumptions of being born in a state of confusion and dualism with the environments, as well as language being a necessary means of the development of the self, have been refuted. There is a wealth of evidence showing that babies are naturally inclined to interact with others, and participation in meaningful social interactions is an important aspect of infancy from the outset. Newborns pay special attention to faces and communicate by crying (Butterworth, 1992, 1995). From around 2 months of age, infants start reciprocating with others, smiling, gazing, and cooing in dyadic face-to-face interactions, and they react to interruptions (such as a sudden “still face”) (Toda and Fogel, 1993). Infants can detect the affordances of emotional expressions at a very young age, provided contextual information is provided (Walker-Andrews and Haviland-Jones, 2005). Further interpersonal skills grow out of transactions with others, such as imitation, joint attention, and communication (Zukow-Goldring, 1997; Rader and Zukow-Goldring, 2012, 2015). The term “primary intersubjectivity,” introduced by Trevarthen (1979), captures the sense of shared pre-linguistic experience and reciprocity. The phenomenological notion of *pre-reflective (non-reflective) self-consciousness* also aims to capture this primary kind of awareness. According to phenomenologists, primary self-awareness does not involve any kind of reflection, introspection, higher-order monitoring, or additional mental state (Zahavi, 2000).

The *implicit self* (Neisser, 1995; Rochat, 2004; Zahavi, 2000; Gallagher and Zahavi, 2023) is a natural consequence of self-related experiences rooted in metamodal direct perception and (inter)action involving the sense of self-agency, self-efficacy, and self-awareness. In this process, there is no logical need to differentiate an ecological self from a social self or to differentiate between the self operating in the social and the non-social realm. There is no need to separate social and non-social aspects of the surrounding environment, as objects and places are inherently social (Costall, 2001). Perception is co-perception, and self-awareness is co-awareness, no matter what aspects of the surrounding environment are foregrounded by an analysis (Rochat, 2004).

This implicit self necessarily exists *in unity*, as argued by Rochat (2019):

“… infants are, from the outset, value-oriented perceivers guided by approach and avoidance skills to specific resources in the environment, and this competence drives and organizes their behavior. Competent, organized behavior is the basis of experiential self-unity. Self-awareness and a primordial and necessary sense of self-unity present early in life is not a result but an initial condition, a “ground zero” of development and learning” (Rochat, 2019, p. 1).

The unity of the self arises out of the multiplicity of experience. As much as Grene concluded that only in a group can one be a highly individualized person, Zahavi claims that “It is only when we are acquainted with a manifold of different acts which are then compared that we can encounter something that is given as the same despite the change in experiences. It is only then that we can encounter something transcendent that retains its identity through changing experiences,” this is how “act-transcendent identity” emerges (Zahavi, 2000, p. 6). Note that this cognitive account involves comparison of separate moments in contrast to the ecological account of the direct perception of persistence and duration.

The unity of the self also implies a focal point of attention: the first-person perspective.^10^ Gibson’s theory (1979, 1966) explains how the first-person perspective is a natural consequence of co-perception of self and environment. Optical flow patterns generated by action provide the agent with a direct awareness of their situated agentive self. Based on this Bermúdez writes: “If the pick-up of self-specifying information starts at the very beginning of life, then there ceases to be so much of a problem about how entry into the first-person perspective is achieved. In a very important sense, infants are born into the first-person perspective. It is not something that they have to acquire *ab initio*.” (Bermúdez, 1998, p. 128, cited by Gallagher and Zahavi, 2023).

Explicit self-awareness emerges by the second year but this development is rooted in and prepared by the development of the implicit sense of self (Neisser, 1995; Rochat, 2004; Butterworth, 1995). More complex forms of language-dependent self-awareness do not take the place of implicit self-awareness throughout the lifespan. Explicit, reflective self-awareness is possible only because there is pre-reflective self-awareness (Gallagher and Zahavi, 2023).

### 3.4 Emergence and rhythm in the development of the ecological self

At this point, we return to the definition of the organism given above to elaborate it in relation to an organicist developmental ecological approach. The definition from Webster and Goodwin (1996) emphasized two aspects of organisms: their development is self-initiated (emergent), and the organism is a life cycle. The first aspect, emergence, is ascribed, in the Modern Synthesis (Neo-Darwinism) to the action and power of the genes, and more behavior-oriented approaches to the ‘interaction’ of organism and environment. Webster and Goodwin eschew both of these approaches and concentrate on morphogenetic fields (1996, pp. 131–257). Our approach is consonant with the field explanations but concentrates on direct perceiving/acting from the point of view of the animal or human organism. The second point, that the organism is a life cycle, we amend. We contend that a cycle entails a circle, whereas all living organisms progress from birth to death, which is not a cycle, that is, a return to the beginning point. Rather, the organism is a life spiral, and animal organisms and human beings are life spirals that live in mutuality with their surrounds, because they directly perceive the layout and events, including people, around them as they live and act.

What changes as an animal or human organism grows, matures, learns, and experiences reorganization? These organisms, as they develop, change two major aspects: first, their locus of activity, and, second, their ‘moment’ of awareness. That is, the animate organism, including humans, is always, when they are awake, co-perceiving self and surround, and they differentiate from infancy on, at least in the case of humans, self-action from other-action or motion. But the locus of their perceiving/acting changes, and sometimes changes radically. The human neonate has a locus of the adult’s body, or of the surface on which they are placed. But when they can crawl, and again when they can walk, this locus becomes radically different--it is transformed. The surfaces available for locomotion change from the immediate flat surface, or the adult body, or a combination of these, to slanted surfaces, objects that are climbable, and so forth. Along with this transformation, other organisms become more peer-like in the possibilities for interaction.

Second, the duration of awareness varies rhythmically with waking/sleeping rhythms, but, also, and more subtly, with changes in the course of life. As an example of the latter, infants anticipate being picked up by an adult as shown in their preparatory movements, but this coordinated action gradually becomes specific to the adults’ actions over the course of the first few months of life (Reddy et al., 2013; Rączaszek-Leonardi et al., 2022). One might say that the older infants could encompass a duration of interaction that included chatting, approaching, and picking up, and could differentiate different aspects of the interaction. Throughout living, the ‘moments’ can become quite extended as invariant aspects of the physical and social surround are perceived. J. Gibson defined familiarity as the feeling a human being has when perceiving invariants over extended events (1979). This perceptually based definition contrasts with the usual definition in social development research in which familiarity is defined as a type of knowledge and a type of ‘stimuli’ (e.g., Rheingold, 1985) (See Read and Szokolszky, 2020; Heft, 2020, on the distinction between stimulation and perceiving). The ecological direct perception account begins with the mutuality of organism and environment, rather than with the need to connect the two. This mutuality is necessary for animals and humans, but it differentiates over the course of the life.

All activity is rhythmic with various durations, and rhythm always involves a return to a previous place or experience, but after having progressed or been changed since the last occurrence. This is a spiral form of living—a return after change, as shown earlier in Figure 5. We note that whereas dynamic systems mathematics has been used as a descriptor of human and animal movement, and of individuals interacting (see above), it is not specific to living organisms but is derived from physics and applies across the living and the nonliving. Living organisms as life cycles (or spirals) that include death are not just physical systems (see DiFrisco and Gawne, 2024, for a critique of the use of dynamic systems to characterize ‘goal directedness’). William Bateson at the turn of the 20th century (see Radick, 2011) proposed that living organisms were a certain type of vortex through which matter cycled. In our view, if vortices are similar to spirals, the vortices of a living organism would have to be distinguished from those of nonliving matter.

## 4 Conclusion

Some consequences of taking the living organism to be the center of biological and psychological developmental research are beginning to be delineated (e.g., Rosslenbroich, 2023). First, the life cycle (or life spiral) is the ‘primary reality of any organism’ (Rosslenbroich, 2023, p. 240). Therefore, „the whole of the organism presents itself completely only in time, not in space. Youth, maturity, and age phases are not present at the same time. Thus, the organism is only partially present as a sensually experienceable object, while the predominant part exists only in its time shape?” (Rosslenbroich, 2023, p. 251). To the extent that one can perceive duration, the organism’s life is perceptible as a set of invariants and variants. Perhaps developing this kind of skilled and differentiated perception is one task of the developmental researcher, in either biology or psychology. The developmental approach to research counteracts the tendency to take the adult form as an objective ‘thing’ because of its relative stability. But to privilege the adult form is to take a’ snapshot’ to be the entirety of a phenomenon (see Rosslenbroich, 2023, p. 240).

We propose that the ecological self is a key to understanding and studying living animal organisms, that is, those with the capacity to perceive and act. The animal organism cannot be completely characterized or understood without taking into account their ability to move and act (which is part of the definition of ‘animal’), and the consequent experiences of self, in the sense of both body and initiator of action, co-existent with surround. It is possible that if we take the ecological self as a focus of developmental research in various topic areas, we will be able to move beyond just comparing abilities at different ages (no matter how much these abilities are seen as part of a ‘system’). Comparing different ages is not much different than comparing different species if we have no way of saying what is constant or continuous about the organism as a life spiral.

The brief review above of ideas regarding the ecological self and unity/awareness of self focused on research in early infancy and the importance of co-perceiving self-and-surround from birth on as the necessary foundation for all later forms of self-awareness. But what form do the changes in the ecological self take over the life spiral? Grene concentrated on the accumulation of experience and the ‘pattern’ that emerges over time in life as being the ‘self’. Could that pattern take the form of a spiral and a spiral that emerges out of a previous spiral?

We have brought up more questions than we have answered, but we intended to point out several possible directions for future Developmental Ecological Psychology research. First, organisms, not abilities, are what develop. Second, organisms are life processes from birth to death. And third, the form of life processes might fruitfully be conceived as a series of spirals that develop out of each other, thus including the fact that life processes are rhythmic, and more complex than cycles. Organisms do not ‘reproduce’, they produce—if they only reproduced, there would be no evolution. Finally, life cycles/spirals must be taken as the basic unit of evolution, as they are the source of development and change throughout life and over generations.

## Data Availability

The original contributions presented in the study are included in the article/supplementary material, further inquiries can be directed to the corresponding author.

## Footnotes

^1^It is important to maintain the distinction between ‘mutuality’ and ‘mutualism’ (we thank David Webster for making this point). Mutuality is a phenomenon—the situated organism acting in and on its surround, perceiving directly. Mutualism is also a phenomenon—the symbiotic living together of two species, but it is also a theory or description of such symbiosis (e.g., the idea of the holobiont, Gilbert and Bard, 2014). Even James Gibson, who carefully developed systematic thinking about mutuality, accepted Darwin’s doctrine of adaptation and, therefore, sometimes made the mistake of thinking about a pre-existing environment as opposed to a ‘surround’ (which cannot exist without an organism). This resulted in such statements as “the environment does not depend on the organism for its existence” (Gibson, 1979, p. 129; as pointed out by Costall, 2001). The theoretical and research work of distinguishing between mutuality and the older concept of mutualism is ongoing (and not always maintained, cf. Costall, 2001).

^2^Mechanists posited that organisms were machines, vitalists invoked an inexplicable life-principle – referred to by the German biologist Hans Driesch as ‘Entelechy’. Entelecheia is the Greek term used by Aristotle; the literal translation of which is ‘being-at-work-staying-itself’ (Sachs, 1995).

^3^“The integration of field forces, hierarchical levels of complexity and gestalt principles into the New Physics promised to make the wholeness of organisms explicable physically and chemically, without reverting to vitalist concepts of an intelligent, controlling life force” (Chichester, 2023, p. 21).This wave of organicism was inspired by post-Newtonian physics, Gestalt psychology and modern art and the ‘recognition that everything is involved in everything else, that the world is not an assemblage of isolated and detachable items but is a continuum of interlocking and mutually dependent activities’ (Chichester, 2023).

^4^We note, for example, that the revised edition of Evolution in Four Dimensions (Jablonka and Lamb, 2006) does not include the topic ‘organism’ in the index. Also, many system-oriented writings on development and evolution do not refer to the organism, e.g., Oyama et al., 2001. There are genotypes and phenotypes, but no organisms.

^5^The organism in mutual relation to its surround is a more specific description than the term ‘organism-environment system’. The former requires direct perception, and the latter does not, although it can be based on direct perception. Also, the system approach does not center the organism, and in some ways diverts attention from the organism. Our thinking on this topic has evolved since 2018 (Read and Szokolszky, 2018a, 2018b).

^6^Several researchers in biology (e.g., Levin, 2019, 2021) attribute ‘selfhood’ even to cells, but that would apply on our view only if the cells were protists with at least qualities of animals.

^7^Our use of the spiral as a model in development contrasts with that of Campill and Valsiner (2023) in that they use Euclidean geometry, and begin with the line. We use descriptive projective geometry (see Field and Gray, 2012) which begins with planes and includes infinity and movement as inherent aspects. “…the inner structure of plane rotation embodies a complex, holistic process of transformation of spatial directions: right becomes left, back becomes front and top becomes bottom” (Elsner, 2013, p. 52). Our aim is to research whether this type of geometric process is helpful in understanding human development over the course of life.

^8^The Ecological Psychology notion of the ecological self is distinct from "the ecology of the self,” as discussed by Hormuth, 1999. In this account "The constituents of the ecology of the self are others, as the sources of direct social experience, objects, as symbols and representations of social experiences, and environments, as the setting for social experiences. They are reflected in self-related cognitions” (p. 2). We also have to note that the term "ecological self” is used in a wholly different sense in environmental philosophy, referring to the self-actualized, ecologically responsible "eco-self” that transcends the "ego-self” (Naess, 1987).

^9^In line with Gibson, Neisser also rejected the postulation of an inner self and maintained that the ecological and interpersonal selves are “unique in being perceived: They need not be recalled, imagined, constructed or conceptualized” (ibid. 3).

^10^In the opposite view, the unity of the self arises out of the multiplicity of experience. In contrast to the direct, duration view of Grene who concluded that only in a group can one be a highly individualized person, Zahavi claims that “It is only when we are acquainted with a manifold of different acts which are then compared that we can encounter something that is given as the same despite the change in experiences. It is only then that we can encounter something transcendent that retains its identity through changing experiences,” this is how “act-transcendent identity” emerges (Zahavi, 2000, p. 6). But on the ecological view experiencing/perceiving does not require or lead to comparison (cf. Pittenger and Dent, 1988).
